# Arrow plot: a new graphical tool for selecting up and down regulated genes and genes differentially expressed on sample subgroups

**DOI:** 10.1186/1471-2105-13-147

**Published:** 2012-06-26

**Authors:** Carina Silva-Fortes, Maria Antónia Amaral Turkman, Lisete Sousa

**Affiliations:** 1 Natural and Exact Sciences Department, Higher School of Health Technology of Lisbon of Polytechnic Institute of Lisbon and Center of Statistics and Applications of University of Lisbon, Lisbon, Portugal; 2 Department of Statistics and Operational Research, Faculty of Sciences of University of Lisbon, and Center of Statistics and Applications of University of Lisbon, Lisbon, Portugal

## Abstract

**Background:**

A common task in analyzing microarray data is to determine which genes are differentially expressed across two (or more) kind of tissue samples or samples submitted under experimental conditions. Several statistical methods have been proposed to accomplish this goal, generally based on measures of distance between classes. It is well known that biological samples are heterogeneous because of factors such as molecular subtypes or genetic background that are often unknown to the experimenter. For instance, in experiments which involve molecular classification of tumors it is important to identify significant subtypes of cancer. Bimodal or multimodal distributions often reflect the presence of subsamples mixtures. Consequently, there can be genes differentially expressed on sample subgroups which are missed if usual statistical approaches are used. In this paper we propose a new graphical tool which not only identifies genes with up and down regulations, but also genes with differential expression in different subclasses, that are usually missed if current statistical methods are used. This tool is based on two measures of distance between samples, namely the overlapping coefficient (OVL) between two densities and the area under the receiver operating characteristic (ROC) curve. The methodology proposed here was implemented in the open-source R software.

**Results:**

This method was applied to a publicly available dataset, as well as to a simulated dataset. We compared our results with the ones obtained using some of the standard methods for detecting differentially expressed genes, namely Welch t-statistic, fold change (FC), rank products (RP), average difference (AD), weighted average difference (WAD), moderated t-statistic (modT), intensity-based moderated t-statistic (ibmT), significance analysis of microarrays (samT) and area under the ROC curve (AUC). On both datasets all differentially expressed genes with bimodal or multimodal distributions were not selected by all standard selection procedures. We also compared our results with (i) area between ROC curve and rising area (ABCR) and (ii) the test for not proper ROC curves (TNRC). We found our methodology more comprehensive, because it detects both bimodal and multimodal distributions and different variances can be considered on both samples. Another advantage of our method is that we can analyze graphically the behavior of different kinds of differentially expressed genes.

**Conclusion:**

Our results indicate that the *arrow plot* represents a new flexible and useful tool for the analysis of gene expression profiles from microarrays.

## Background

Genome-wide expression analysis is an increasingly important tool for identifying gene function, disease-related genes and transcriptional patterns related to drug treatments. Microarrays enable the simultaneous measurement of the expression levels of tens of thousands of genes and have found widespread application in biological and biomedical research. Increasing numbers of multi-class microarray studies are performed, but the vast majority continues to be two class (binary) studies, for example when both control and a treatment are examined [1-4]. The objective of the study in most of them, is to determine the genes that are differentially expressed between the two classes. Differentially expressed genes are usually detected using statistics based on means or medians. However, if there are genes differentially expressed on different subclasses, those techniques do not select them because either mean or median values tend to be similar between the considered groups.

Genes with a bimodal or a multimodal distribution within a class (considering a binary study) may indicate the presence of unknown subclasses with different expression values [5,6], meaning that there are two separate peaks in the distribution; one peak due to a subclass clustered around a low expression level, and a second peak due to a subclass clustered around a higher expression level. As a consequence, the identification of such subclasses may provide useful insights on biological mechanisms underlying physiologic or pathologic conditions. In cancer research, a common approach for prioritizing cancer-related genes is to compare gene expression profiles between cancer and normal samples, selecting genes with consistently higher expression levels in cancer samples. Such an approach ignores tumor heterogeneity and is not suitable for finding cancer genes that are overexpressed in only a subgroup of a patient population. As a result, important genes differentially expressed in a subset of samples can be missed by gene selection criteria based on the difference of sample means [7].

The particular application that motivated our work concerns the development of a methodology which could simultaneously identify up- and down-regulated genes and differentially expressed with bimodal or multimodal distributions with similar means on both groups. For convenience, the latter case is referred to as *special genes*.

Different statistical tests have been proposed to select differentially expressed genes [8-11]. Among them, is the receiver operating characteristic (ROC) analysis, which is widely used to evaluate a diagnostic system but can be interpreted as a measure of separation between two distributions.

**Figure 1:**
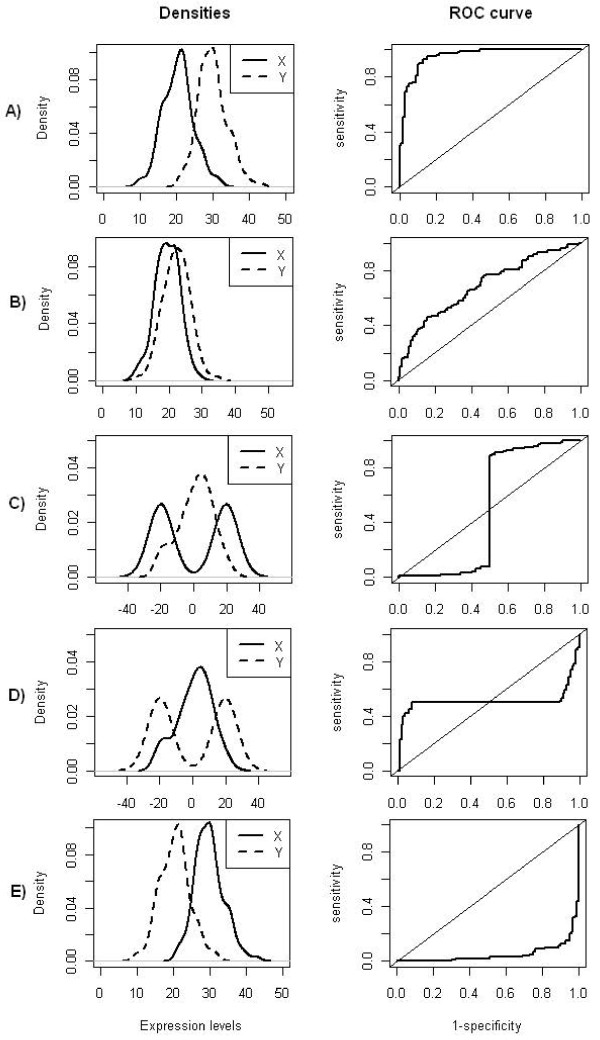
**Relationship between densities and ROC curves considering equal variances on both groups.** Probability density functions of gene expression values of two groups and their corresponding empirical ROC curves, where *Y * is the random variable which represents the expression values under the experimental condition and *X* the random variable which represents the expression values for the control group. The same classification rule was considered for all ROC plots, namely, high values of the decision variable correspond to positive regulation. Density plots were obtained using kernel density estimation from two samples of size 100 simulated from normal distributions. **A)***X* ∼ *N*(20, 4),*Y* ∼ *N*(30, 4); **B)***X* ∼ *N*(20, 4),*Y* ∼ *N*(22, 4); **C)***X* ∼ 0.5*N*(−20, 2) + 0.5*N*(20, 2),*Y* ∼ *N*(0, 11); **D)***X* ∼ *N*(0, 11),*Y* ∼ 0.5*N*(−20, 2) + 0.5*N*(20, 2); **E)***X* ∼ *N*(30, 4),*Y* ∼ *N*(20, 4).

A ROC curve displays the relationship between the proportion of true positive (sensitivity) and false positive (1-specificity) classifications resulting from each possible decision threshold value in a two class classification task [12]. These proportions depend on the classification rule and in general higher values of the marker are associated with the case group. However, if ROC analysis is blindly applied to select genes differentially expressed, i.e., keeping the same classification rule for all genes in an experiment, not proper ROC curves (NPROC) [11] can be produced because genes with positive and negative regulation have opposite classification rules. NPROC curves are obtained when they cross or are below of the reference line (Figure 1C–E).

Genes can be ranked using the area under the ROC curve (AUC) [10,11], a common measure of discrimination, which should range between 0.5 and 1, but for NPROC curves AUC can have values below 0.5.

**Figure 2:**
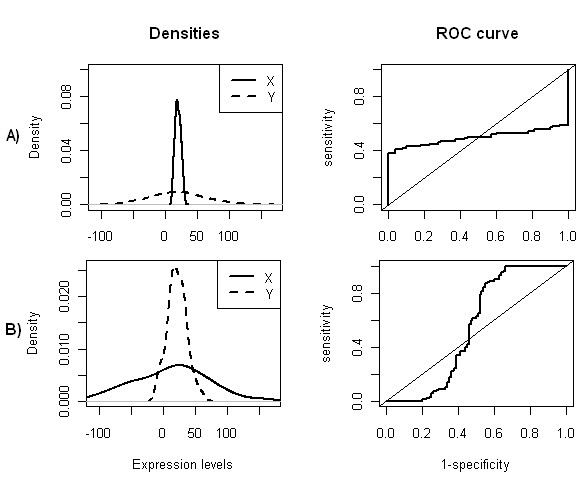
**Relationship between densities and ROC curves, considering different variances and similar means on both groups.** Probability density functions of gene expression values of two groups and their corresponding empirical ROC curves, where *Y * is the random variable which represents the expression values under the experimental group and *X* is the random variables which represents the expression values for the control group. The same classification rule was considered in all ROC plots, i.e., high values of the decision variable correspond to positive regulation. Density plots were obtained using kernel density estimation from two samples of size 100 simulated from normal distributions. **A)***X* ∼ *N*(20, 15),*Y* ∼ *N*(20, 60); **B)***X* ∼ *N*(20, 40),*Y* ∼ *N*(20, 5).

Nevertheless, different scenarios can lead to NPROC curves, for instance, when the means of the two groups are similar and one of the groups has a bimodal distribution (Figure 1C–D) (or multimodal), or when both distributions are unimodal with similar means and significant different variances (Figure 2). On both cases the corresponding ROC curve will have a sigmoidal-shape with an AUC close to 0.5.

Proper binormal model [13] and contaminated binormal model [14] are methods that force the ROC curves to be set above the reference line when they are not proper and consequently the AUC will be higher than 0.5. However in the context of this work, not proper ROC curves have an important role in the selection of different kinds of differentially expressed genes.

Since it is not possible to decide beforehand the direction of the classification rule, we considered the same classification rule for all of the genes, i.e., values of expression levels above the threshold correspond to up-regulation. In that sense, AUC values near 1 will correspond to up-regulated genes, AUC values near 0 will correspond to down-regulated genes, and special genes (Figure 1C–D) will have an AUC around 0.5. However, regardless of the type of distributions, if means are similar (Figure 1B, Figure 2), AUC will be near 0.5. So, using AUC is not sufficient to select special genes.

We used the overlapping coefficient (OVL) to further separate these different situations which produce values of AUC near 0.5. Bradley [15] and Inman and Bradley [16] promote the use of OVL as an intuitive measure of the similarity between two probability distributions. Graphically, OVL is the area where the densities of the two distributions overlap when plotted on the same axes.

We propose using AUC and OVL simultaneously to select different types of differentially expressed genes and plotting OVL against AUC we get a picture which we named as *arrow plot*.

If we consider that groups have different variances, special genes can be mixed with genes which are not differentially expressed as illustrated on Figure 2, that is, genes with unimodal densities, with similar means but significantly different variances. These genes will have AUC values around 0.5 and low OVL values. With the purpose of identifying genes under these conditions, allowing their separation from the special genes, we developed an algorithm based on finding bimodality (or multimodality) using kernel densities estimates.

Nonparametric techniques are used to estimate AUC and OVL. To estimate AUC, we used the Mann-Whitney U statistic [17], which is equivalent to the trapezoidal rule for integration. For the OVL, we developed an algorithm where a naive kernel density estimator [18] is used to construct a nonparametric estimator of OVL.

We first describe the algorithm and later we evaluate the performance of our method by comparing the gene expression profiles in two different classes using data from a publicly dataset [6] and from a simulated dataset. The first dataset consists of 14 different samples of normal circulating B cells (controls) and 20 heterogeneous lymphomas (experimental group) [6]. The gene expression data were obtained on 4026 genes. The simulated dataset consists of 10000 genes generated from a lognormal distribution, where each group sample has 30 arrays. Using publicly available data, we compared our results with those obtained by Parodi et al. [11] using as methods, the area between the ROC curve and rising diagonal (ABCR) and a test for not-proper ROC curves (TNCR). We used both data sets to assess the relative performance of our proposed method as compared to the most common different statistical gene ranking measures.

All the analysis were performed using the open-source R software [19] and packages from Bioconductor [20].

## Results and Discussion

### Algorithm description

**Figure 3:**
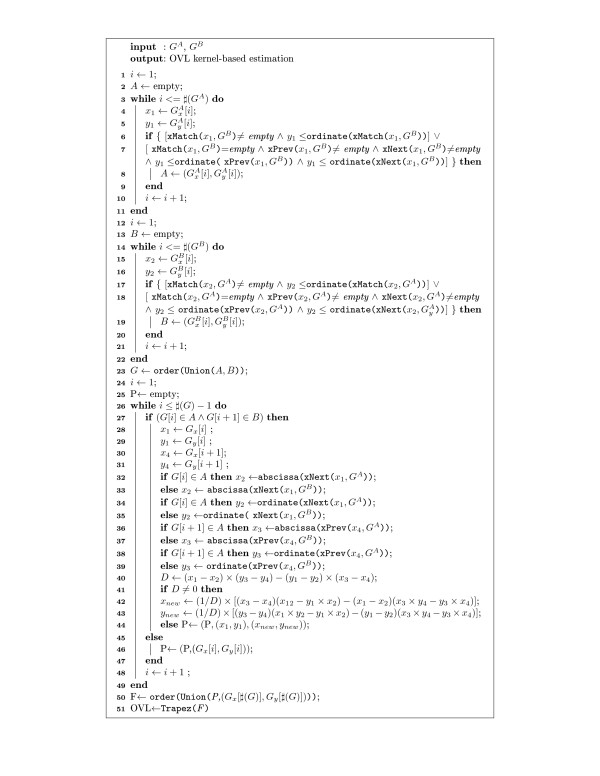
**Algorithm 1.** Pseudo code to estimate OVL based on kernel density estimates.

**Figure 4:**
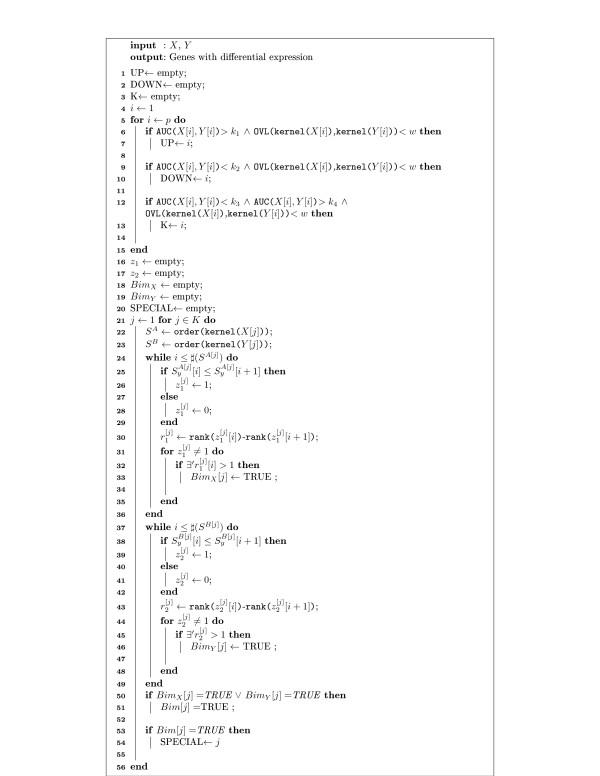
**Algorithm 2.** Pseudo code to select differentially expressed genes based on AUC and OVL estimates.

For illustrative purposes, we divided the algorithm in two parts (algorithm 1 and algorithm 2). The first part describes the OVL estimation (Figure 3) and the second part describes the selection of different kinds of differentially expressed genes (Figure 4).

The OVL estimation was based on a non-parametric form with densities estimated using kernel functions. Figure 3 shows the pseudo-code which implements the OVL estimation and Tables 1 and 2 describe the notation and functions used there. The OVL values are computed by finding the points that belong to the area of intersection of the two densities (Figure 3: lines 1–21) and the jump points between densities, which are estimated by interpolation (Figure 3: lines 24–44). The points are combined into one set and sorted in ascending order (Figure 3: line 50). Finally OVL is estimated using a trapezoidal rule considering a non-uniform grid-spacing (Figure 3: line 51).

**Table 1:** List with the notation used in Algorithm 1

**Symbol**	**Definition**
*G*^*A*^, *G*^*B*^	Kernel density coordinates of samples A and B
*A*, *B*	pairs of coordinates of samples A and B that will be used
	to estimate OVL
$G_{x}^{A}[i],G_{y}^{A}[i]$	index a pair of coordinates of *G*^*A*^,
	where $G_{x}^{A}[i]$ is the abscissa and
	$G_{y}^{A}[i]$ is the correspondent ordinate (same to *G*^*B*^ and *G*)
*♯*(.)	total number of pairs of coordinates
*G*	ordered list of points resulting from the union of *A* and *B*
*P*	union of G with new pairs of coordinates,
	which correspond to jump points between densities
*G*[*i*]	indexes a pair of coordinates of G
*x*_*new*_	new abscissa
*y*_*new*_	new ordinate
*F*	final list of pairs of coordinates to estimate OVL
*OVL*	overlapping coefficient between two kernel densities

Symbols are listed in order of appearance in the Algorithm.

**Table 2:** List of functions used in Algorithm 1

**Function**	**Definition**
xMatch	if there is more than one equal abscissa
(abscissa,list)	on the list, returns the pair of
	coordinates corresponding to the
	one which has the minimum ordinate
ordinate	returns the ordinate of a pair of
(abscissa,ordinate)	coordinates
xPrev	returns the pair of coordinates immediately
(abscissa,list)	preceding the abscissa in the list
xNetx	returns the pair of coordinates immediately
(abscissa,list)	after the abscissa in the list
Union(list,list)	joins lists
order(list)	orders a list in increasing order of abscissas
abscissa	returns the abscissas of a pair of
(abcissa,ordinate)	coordinates
trapez(list)	trapezoidal rule for area estimation

Functions are listed in order of appearance.

The selection of differentially expressed genes is based on simultaneous analysis of OVL and AUC. The *arrow plot* is obtained by plotting OVL on abscissas and AUC on ordinates. Figure 4 shows the pseudo-code which implements the algorithm to select differentially expressed genes based on these two measures and Tables 3 and 4 present the notation and functions used there.

**Table 3:** List with the notation used in Algorithm 2

**Symbol**	**Definition**
*X*	*p* × *n* matrix, corresponding to sample A with columns
	representing arrays and rows representing genes
*Y*	*p* × *m* matrix, corresponding to sample B with columns
	representing arrays and rows representing genes
UP	up-regulated genes list
DOWN	down-regulated genes list
*X*[*i*], *Y*[*i*]	indexes a gene (row of the matrix)
*k*1, *k*2, *k*3	arbitrary thresholds
*k*4, *w*	
*S*^*A*^, *S*^*B*^	kernel density coordinates of subsamples of genes from
	samples A and B
*S*^*A*[*j*]^	indexes a gene of the subsample S from sample A
$S_{y}^{A[j]}[i]$	indexes a ordinate of a gene j of the subsample S from
	sample A
*Bim*_*X*_[*j*]	indexes a gene with bimodal or multimodal kernel density
	from sample A
*Bim*[*j*]	indexes a gene with bimodal or multimodal kernel density
SPECIAL	special genes list

Symbols are listed in order of appearance.

**Table 4:** List of functions used in Algorithm 2

**Function**	**Definition**
AUC(list,list)	Area above the ROC curve
	estimated by the trapezoidal rule
OVL(list,list)	overlapping coefficient estimated by Algorithm 1
kernel(list)	kernel density estimation
rank(list)	returns the ranks of a list

Functions are listed in order of appearance.

Selection of differentially expressed genes with positive regulation (Figure 4: line 6–7) and negative regulation (Figure 4: line 9–10), is made according to arbitrarily selected cutoff points for AUC and OVL. However, AUC values are expected to be close to 1 for up-regulated and close to 0 for down-regulated genes and OVL will have low values on both situations. Selection of special genes is performed in two steps. The first step consists on the selection of genes with AUC values near 0.5 and low values of OVL (Figure 4: lines 12–13). Since the variances on both groups can be different, it is possible to find genes with no-differential expression mixed with the special ones. Accordingly, the second step aims at removing the genes without differential expression, through the bimodality analysis.

Bimodality (or multimodality) is analyzed based on the behavior of the ordinates of the kernel based estimated densities of both groups, considering only the gene list that is selected in the first step mentioned above (Figure 4: line 13). The points of both densities are ordered in increasing order of abscissas (Figure 4: lines 22–23). If an ordinate is equal or less than the ordinate immediately after, it is assigned a label 1 and 0 otherwise (Figure 4: lines 25–28 and 38–41). This allows us to analyze the variation of the density over the observed range. Considering only the points where the function is increasing, if the differences between the ranks of adjacent ordinates is 1, the distribution is expected to be unimodal, otherwise the distribution will be bimodal or multimodal (Figure 4: lines 30–33 and lines 43–46). To declare a gene to be special it is enough to find bimodality in one of the groups (Figure 4: lines 50–54), yet it is of interest to analyze in which group bimodality is observed, and this is possible using different color labels on the *arrow plot*.

#### Performance and implementation

The running time of the algorithm in a dataset with 10000 genes, takes less than 60 minutes on a 533 MHz Pentium.

R source code for the implementation of this algorithm is available in Additional file 1.

#### Lymphoma data

**Figure 5:**
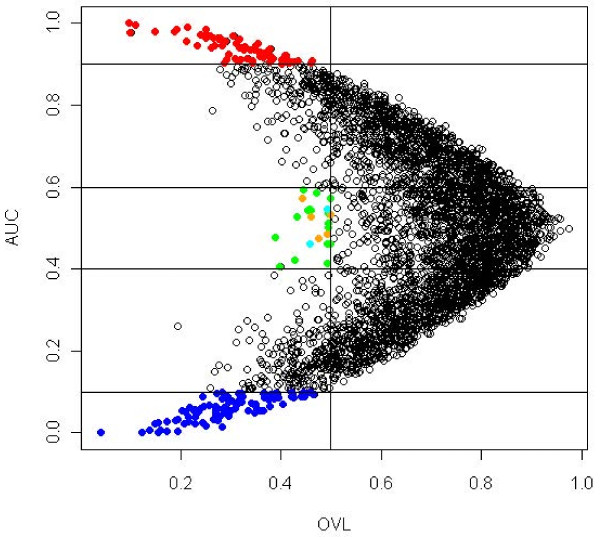
**Arrow plot of lymphoma data.** AUC≥ 0.9 and OVL< 0.5 was considered to select up-regulated genes, corresponding to red dots on the plot. To select down-regulated genes an AUC≤0.1 and OVL< 0.5 was considered, corresponding to blue dots on the plot. To select special genes an OVL< 0.5 and 0.4 <AUC< 0.6 was considered. Orange dots correspond to a bimodal or multimodal density in the experimental group, cyan dots correspond to a bimodal or multimodal density in the control group and green dots correspond to a bimodal or multimodal densities in both groups.

From a total of 4026 genes, our method selected 178 differentially expressed genes, where 68 corresponded to up-regulated genes, 90 to down-regulated and 20 corresponded to special genes. We used AUC≥0.9 and OVL<0.5 to select up-regulated genes, AUC≤0.1 and OVL<0.5 to select down-regulated genes and OVL<0.5 and 0.4<*AUC*<0.6 to select special genes. Thresholds were chosen arbitrarily, although an analysis of the the *arrow plot* (Figure 5) could help on deciding which thresholds to use.

**Figure 6:**
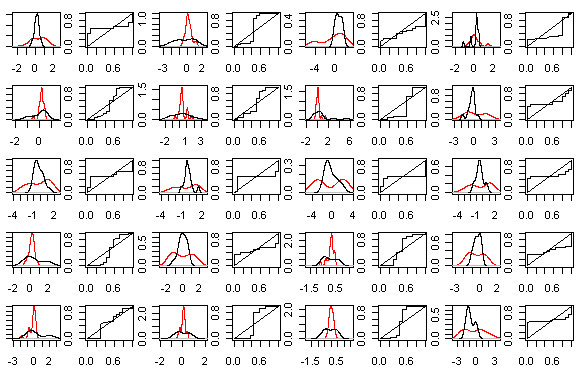
**Kernel density plots and empirical ROC plots.** Kernel density estimate of the 20 special selected genes expression values, where red densities represent the experimental sample and black densities represent the control sample. The *x*-axis is on log base 2 scale. From left to the right, each plot pair correspond to densities and respective empirical ROC curve of the gene ID’s: GENE1141X GENE3521X, GENE3547X, GENE3473X, GENE2547X, GENE2519X, GENE1877X, GENE3343X, GENE3322X, GENE3323X, GENE3389X, GENE3388X, GENE3909X, GENE2887X, GENE2778X, GENE463X, GENE1004X, GENE3407X, GENE75X, GENE1817X.

Table 5 shows the 20 selected special genes. Genes are listed in ascending order of OVL, which ranged between 0.389 and 0.499. AUC values ranged between 0.407 and 0.593. Bimodality was tested on the 20 special genes; 2 genes have bimodality in the control group, 5 genes on the experimental group and 13 genes on both. For the 20 special genes, kernel densities and their corresponding empirical ROC curves can be analyzed in Figure 6. All the selected genes had a sigmoidal-shaped ROC curve.

**Table 5:** AUC and OVL values and bimodality group identification of the 20 selected special genes

**Gene ID**	**Gene name**	**OVL**	**AUC**	**Group**
GENE3323X	*BCL7A*	0.389	0.477	B
GENE3473X	*Unknown*	0.399	0.407	B
GENE1877X	*Unknown*	0.428	0.421	B
GENE3388X	*Immunoglobulin J chain*	0.432	0.529	B
GENE1141X	*MAPKKK5*	0.443	0.571	E
GENE3521X	*Similar to KIAA0050*	0.446	0.593	B
GENE3407X	*Histone deacetylase 3*	0.453	0.543	B
GENE75X	*VRK2 kinase*	0.457	0.546	C
GENE2519X	*Unknown*	0.461	0.529	E
GENE3343X	*LR11*	0.461	0.543	B
GENE1817X	*BL34*	0.472	0.586	B
GENE3389X	*Immunoglobulin J chain*	0.476	0.475	E
GENE3909X	*Placental bikunin*	0.492	0.463	C
GENE2887X	*LBR*	0.492	0.486	E
GENE3547X	*Immunoglobulin kappa*			
	*light chain*	0.493	0.413	B
GENE1004X	*BNIP3*	0.494	0.511	B
GENE2547X	*CLK3 kinase*	0.495	0.500	B
GENE2778X	*DNA Ligase III*	0.496	0.536	B
GENE3322X	*BCL7A*	0.498	0.532	E
GENE463X	*PARP*	0.499	0.461	B

Special genes were selected using OVL<0.5 and 0.4<*AUC*<0.6. E: bimodality in experimental group, C: bimodality in control group and B: bimodality in both groups. Genes are ordered by ascending order of OVL.

Among the 20 special genes selected list (Table 5), 3 have an unknown regulatory function. All the remaining 17 genes are related with proteins encoding. GENE3323X (*BCL7A*) and the GENE3388X (*Immunoglobulin J chain*) are presented in other clones in the same dataset, GENE3322X and GENE3389X respectively. Alizadeh et al. [6] observed that *BCL7A* gene can be altered by translocation in lymphoid malignancies. The biological properties of the 20 selected genes are described in the Additional file 2.

We compared our results with those obtained by Parodi et al. [5], where ABCR and TNRC statistics were used. According to the highest TNRC value, a total of 1607 differentially expressed genes were considered, and 16 of them were special genes. Eight of them are considered to be special according to our methodology. The remaining 8 genes of their list have AUC and OVL values slightly higher than the considered cutoff points on our study. However, if we choose threshold values for AUC and OVL to catch those genes, we will select 85 more special genes.

Nine feature selection methods were applied to the full dataset, namely Welch t-statistic, fold change (FC), rank products (RP) [21], average difference (AD) [22], weighted average difference (WAD) [23], moderated t-statistic (modT) [24], intensity-based moderated t-statistic (ibmT) [25], significance analysis of microarrays (samT) [26] and area under the ROC curve (AUC). We assessed the overlap between gene lists produced by different feature selection methods and ranked lists of differentially expressed genes were produced. We examined the top 20 mostly highly ranked genes, and for all methods the 20 special genes selected by our methodology are missed.

#### Simulated data

**Figure 7:**
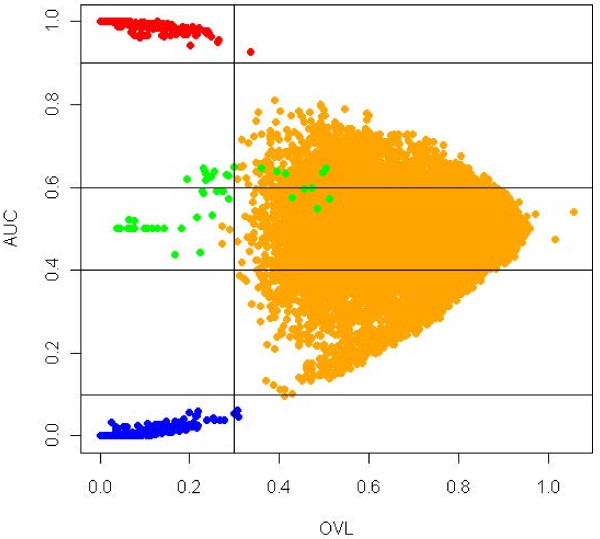
**Arrow plot of simulated data.** Orange dots correspond to truly no differentially expressed genes, red dots correspond to truly up-regulated genes, blue dots correspond to truly down-regulated genes and green dots to truly special genes. We considered as up-regulated genes those for which AUC≥ 0.9 and an OVL< 0.5. To select down-regulated genes an AUC≤ 0.1 and an OVL< 0.5 were considered and to select differentially expressed genes with bimodal or multimodal densities we considered an OVL< 0.5 and 0.4 <AUC< 0.6.

We simulated ten thousand genes (see Methods for details), among which 9500 were non-differentially expressed, 225 were up-regulated, 225 were down-regulated and 50 were special genes. Analyzing the *arrow plot* (Figure 7), we considered 0.3 as threshold value for the OVL. As for the AUC, we classified as up-regulated those genes with AUC above 0.9, as down-regulated genes those with AUC below 0.1 and special genes those with AUC between 0.4 and 0.6. In the *arrow plot* we can observe the distribution of the truly 500 differentially expressed genes, and we can conclude that 95% of them were selected by our methodology. In the first step of the algorithm used to select special genes (Figure 4), 33 genes which were candidate to special genes were selected. Through the second step we found that all of the genes had bimodality in at least one of the groups.

We can conclude that our algorithm for detection of bimodality performed with 100% of accuracy on that list.

ROC analysis was conducted to evaluate and compare the performance of the above methods. We analyzed the performance of these methods regarding the discrimination between differentially expressed genes and non-differentially expressed genes considering two scenarios. First we studied the performance of the methods concerning the capacity to differentiate among up-regulated, down-regulated and special genes; secondly we studied the performance concerning only the capacity to identify special genes.

The construction of the ROC curves were based on the absolute values of the following statistics: FC, AD, WAD, RP, Welch-*t*, SAM, SAMROC, ibmT, modT and shrinkT, where high values are related to DE genes. The ROC curve for the AUC method was constructed considering AUC values ranging from 0.5 to 1; in this way, any AUC value below 0.5 was substituted by its complementary value, i.e., by 1−AUC. High AUC values are related to DE genes. When analyzing the *arrow plot*, we verified that only the OVL statistic is needed since lower values of the OVL correspond to DE genes.

**Figure 8:**
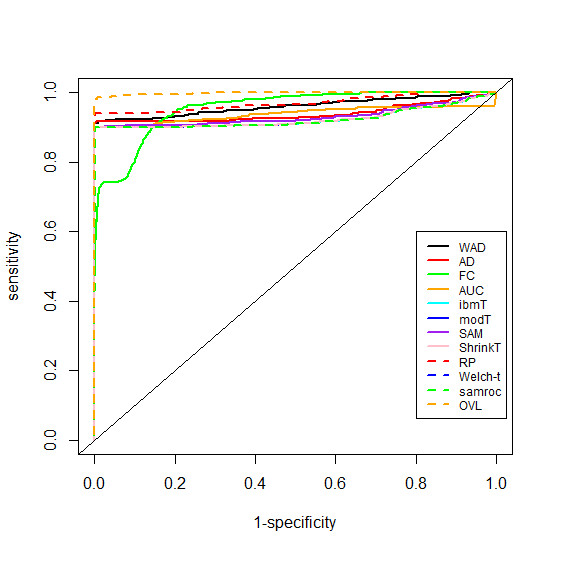
**Empirical ROC curves.** Comparison of ROC curves in experiments where the goal is to select up- and down-regulated genes and special genes.

The empirical ROC curves, under the first scenario are represented in Figure 8, and the respective empirical AUC values are displayed on Table 6.

**Table 6:** Empirical AUC values

**OVL**	**RP**	**WAD**	**FC**	**AD**	**AUC**
0.998	0.969	0.959	0.953	0.939	0.937
**SAM**	**ibmT**	**modT**	**Welch-*t***	**ShrinkT**	**SAMROC**
0.930	0.924	0.924	0.924	0.924	0.921

Comparison of AUC values where the goal is to select up- and down-regulated genes and special genes. The AUC values are sorted by decreasing order.

The OVL with an estimated AUC value near of the unit showed to be the one with a better performance followed by the Rank Products method. The method with lowest performance was SAMROC, however all methods showed high values of performance.

**Figure 9:**
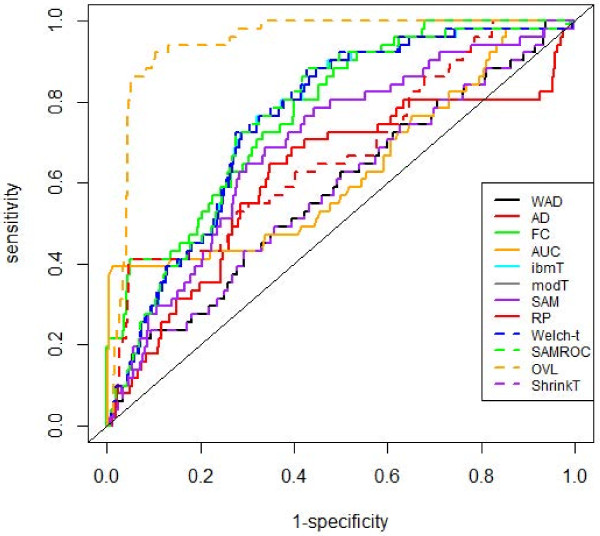
**Empirical ROC curves.** Comparison of ROC curves in experiments where the goal is to select special genes.

Considering the scenario where the goal is to select only special genes, the empirical ROC curves (Figure 9) and the empirical AUC values (Table 7) showed that OVL was the method with better performance followed by the FC method, however with an AUC value considerably low. WAD and shrinkT were the methods with the lowest performance.

**Table 7:** Empirical AUC values

**OVL**	**FC**	**SAMROC**	**Welch-t**	**ibmT**	**modT**
0.9459	0.7786	0.7608	0.7604	0.7555	0.7545
**SAM**	**RP**	**AUC**	**AD**	**WAD**	**shrinkT**
0.6934	0.6733	0.6288	0.6140	0.5793	0.5793

Comparison of AUC values where the goal is to select special genes. The AUC values are sorted by decreasing order.

## Conclusions

We have presented a graphical and computational method for microarray experiments which allow the identification of genes that express differently under two conditions even if the behavior in average is similar. The main objective of this work was to select differentially expressed genes due to the presence of different subclasses, which could give important information about their inherent biological functions, and that are usually missed by usual methods.

AUC and OVL statistics were used to achieve this goal. Both statistics are invariant when a suitable common transformation is made on variables [12,16], and on microarray data analysis log transformations are widely used. *Arrow plot* is obtained by plotting OVL against AUC. This plot is easily interpreted because both statistics range between 0 and 1, and in addition to detecting genes with up- or down-regulation, *arrow plot* is also able to detect special genes, however for the latter genes a bimodality analysis needs to be added.

The approach used by the *arrow plot* is similar to the volcano plot, in the sense that two selection criteria are needed to select genes. Using double filtering criterion will obtain a more robust result. Yet, the cost we pay is that some true differentially expressed genes might be missed. However, *arrow plot* allows us to pick some genes from the single filtering region for further examination.

Non-parametric techniques were used because they eliminate the need to specify parametric models. The non-parametric kernel density method has few assumptions about the form of the distributions. This is attractive because it can be used on thousands of genes on an automatic way. The disadvantage of non-parametric techniques is that it results in a loss of efficiency. Yet, the loss of efficiency is balanced by the reduction of the risk of misinterpreting the results by incorrectly specifying a parametric form for the distribution.

The proposed algorithm is particularly useful in situations where bimodality exists in the gene expression data. The proposed methodology outperforms other well known methods for detecting different kinds of differentially expressed genes. Future work includes further evaluation of this methodology on other real datasets.

We recognize that selecting DE genes through an *arrow plot* has shortcomings. For instance, using arbitrary cut-off points for AUC and OVL will require the user to have some experience and results are sensitive to the cut-off choice. Nevertheless, the analysis of the *arrow plot* will help the user to select the cut-off points for AUC and OVL. This plot has to be seen as a statistical exploratory tool rather than an inference tool. The objective of the plot is the visual identification of genes which can play a special role. No other plot is able to achieve this goal.

*Arrow plot* is an exploratory graphical tool for microarray experiments, useful in the identification of different kinds of differentially expressed genes, particularly in the identification of genes with a special behavior which are not detected by usual methods and yet can bring relevant biological information. This methodology can be used in all platforms.

## Methods

### Data sets

#### Lymphoma data

We used microarray data provided by the study of Alyzadeh et al. (2000) [6] which are publicly available at the website http://llmpp.nih.gov/lymphoma/data/figure1/. They used a special microarray called Lymphochip, where they selected genes that are preferentially expressed in lymphoid cells and genes with known or suspected roles in important processes in immunology or cancer. They used these microarrays to characterize gene expression patterns in the three most prevalent adult lymphoid malignancies: DLBCL (diffuse large B-cell lymphoma), FL (follicular lymphomas) and CLL (chronic lymphocytic leukemia). They also profiled gene expression in purified normal lymphocyte subpopulations under a range of activation conditions (see original paper for more details [6]). Fluorescent cDNA probes, labelled with the Cy5 dye, were prepared from each experimental messenger RNA sample. A reference cDNA probe, labeled with the Cy3 dye, was prepared from a pool of mRNAs isolated from nine different lymphoma cell lines. Each Cy5-labelled experimental cDNA probe was combined with the Cy3-labelled reference probe and the mixture was hybridized to the microarray. The fluorescence ratio was quantified for each gene and reflected the relative abundance of the gene in each experimental mRNA sample compared with the reference mRNA pool. The ratio values were log transformed (base 2) and stored in a Table (rows, individual cDNA clones; columns, single mRNA samples). The dataset that we used in our study is part of the original one, and was the same used in the study of Parodi et al. [5]. This database included 4026 gene expression profiles, where the control group had 14 samples of normal circulating B cells (NBC), of which 6 were highly stimulated and 8 slightly or not stimulated samples. The experimental group had 20 heterogeneous lymphomas by pooling 9 samples of FL and 11 samples of CLL. Both classes included two subclasses, namely: 6 heavily stimulated and 8 slightly stimulated or unstimulated samples in controls and 9 follicular lymphomas and 11 chronic lymphocytic leukemias in experimental group. Parodi et al. [5] estimated missing data by the method proposed by Troyanskaya et al. [22].

#### Simulated data

We conducted a simulation study in order to evaluate the performance of the proposed method.

Most studies of microarray data assumed normality assumptions. However, there is relatively little literature on evaluating the normality of this type of data. Part of the problem is that most microarray datasets include large amounts of biological variability and/or small sample sizes. Biological variability makes it difficult to determine the source of the non-normality (non-normal datasets could simply be mixtures of normal datasets). Small samples do not have the power to be able to make claims about the distribution of the data.

It is well known that raw microarray data (across all platforms) are highly skewed (usually skewed right) with many extreme values, so, simulated datasets were generated by drawing case and control samples from lognormal distributions, and log transformation was used afterwards to offset the skewness. Consider *X* a random variable representing the expression levels in the control sample, where *X* ∼ *logN*(*μ*_*x*_, *σ*_*x*_) and *Y* a random variable which represents the expression levels in case sample, where *Y* ∼ *logN*(*μ*_*y*_, *σ*_*y*_).

For case and control samples we simulated *n*_1_ = *n*_2_ = 30 microarrays and a total of 10000 genes. This sampling was performed independently, albeit the fact that individual gene expression levels are far from being independent. In a typical microarray experiment, we expect to see a combination of non-differentially and differentially expressed genes (approximately 5% to 10% of the data). Hence, we simulated 500 genes differentially expressed and 9500 not differentially expressed. From the 500 differentially expressed genes, 225 were up-regulated, 225 were down-regulated and 50 corresponded to special genes.

Four characteristics of the data were considered in this simulation: mean (*μ*), variance (*σ*^2^), the magnitude of difference between control and case samples and bimodality of the distributions. Hence, several combinations of these parameters were considered.

While simulating values for expression levels of genes not differentially expressed, we considered that the difference between the mean of the control and case arrays ranged between -0.9 and 0.9. To provide several patterns of density distributions we considered variances with differences ranging from 0 and 12.25. The effect of changing *σ* does not seem to affect these genes because all arrays came from the same nearly mean vector. However, some of these genes will be mixed with the special ones when the variances between case and control samples are significantly different.

Genes with up-regulation and down-regulation were generated considering the difference between the mean of the case and control arrays ranging from 3.5 to 13.5 for up-regulation, and -13.5 to -3.5 for down-regulation and the differences between the variances for both situations ranged from 0 to 12.25.

Gene expression distribution of a special gene was considered as a mixture of two lognormal distributions in one of the groups. If *X* is a random variable following this distribution, we write $X∼\Theta_{\alpha ,\mu_{0},\sigma_{0},\mu_{1},\sigma_{1}}$ with the distribution defined by *α *log*N*(*x*;*μ*_0_,*σ*_0_) + (1 − *α*)log*N*(*x*; *μ*_1_, *σ*_1_),*x* > 0, where logN(x;μ,σ) denotes a lognormal distribution with location and scale parameters *μ* ∈ *ℜ* and *σ* > 0, respectively, and *α* ∈ (0, 1) specifies the contribution to the total of the two single lognormal components. The parameters *μ* and *σ* become the mean and the standard deviation of the normal distribution upon log transformation of the lognormal random variable. To simulate special genes we considered bimodality in one of the groups. For the mixture we left *μ*_0_ = 3.5 unchanged and gradually increased *μ*_1_ from 7 to 17, and left *σ*_0_ = *σ*_1_ = 1.2 unchanged. For the other group we considered a lognormal density with location parameter approximately equal to *α* × *μ*_0_ + (1 − *α*) × *μ*_1_. We considerer *α* = 0.5.

Finally we took the logarithms of the 10000 expression levels on both groups to offset the skewness.

### Non-parametric OVL

The overlapping coefficient refers to the area under two density functions simultaneously [15]. OVL is formally defined by (1):

(1) $$\;\text{OVL}=\underset{c}{\int }\text{min}\;[f_{X}(c),f_{Y}(c)]\;\text{dc},$$

where *f*_*X*_and *f*_*Y*_ are the density functions of the random variables *X* and *Y * respectively. The results are directly applicable to discrete distributions by replacing the integral with a summation.

The estimation of OVL was based on a non-parametric procedure with densities estimated using kernel functions. A kernel function *K*(.) is defined as a continuous, limited and symmetric function, with the property that its indefinite integral is equal to unity, ∫ *K*(*u*)*du* = 1. The typical form of a kernel density estimator is given by (2):

(2) $$\;\hat{f}(x)=\frac{1}{nh}\overset{n}{\underset{i=1}{\sum }}K\left(\frac{x-x_{i}}{h}\right),$$

where *h* is the bandwidth, known as the shaping parameter and (*x*_1_,…,*x*_*n*_) is the sampling vector.

For the purpose of this work, we chose as kernel function a standard normal distribution ${\left(2\Pi \right)}^{-\frac{1}{2}}\text{exp}(-\frac{1}{2}u^{2})$.

More than the choice of the kernel function, the choice of the bandwidth, *h*, is what drives the kernel estimator. A choice of the bandwidth *h* satisfying some optimal criteria [27], is given by (3):

(3) $$\;h={\left(\frac{4}{3}\right)}^{\frac{1}{5}}sn^{-\frac{1}{5}},$$

where *s* is the empirical standard deviation.

However this choice of *h* may tend to over-smooth the distribution if the population is multimodal. A better result may be obtained by using the interquartile range, *R*[28]. If the distribution of interest is bimodal, using interquartile range may over-smooth further. Therefore the use of an adaptive measure of spread is recommended (4):

(4) $$\;h={\left(\frac{4}{3}\right)}^{\frac{1}{5}}\;\text{min}\;\left(s,\frac{R}{1.34}\right)\;n^{-\frac{1}{5}}.$$

The function density from R uses as default settings the normal kernel and the value for *h* given in (4). In our calculations of the OVL, we used this function from R, with the default settings to estimate the densities, and for the trapezoidal rule needed to computed the area, we used the function trapz of the library caTools from R.

### Non-parametric AUC

ROC curve assesses the effectiveness of a continuous diagnostic marker in distinguishing between two independent populations. In a standard situation a case is assessed positive if the corresponding marker value is greater than a given threshold value. Associated with any threshold value is the probability of a true positive (sensitivity) and the probability of a true negative (specificity). Let *X* be the random variable for the marker on the control group and *Y * the random variable for the marker on the case group. For any given threshold value *c*, sensitivity is given by *P*(*Y* >*c*) = 1 − *F*_*Y*_(*c*), and specificity is given by *P*(*X* ≤ *c*) = *F*_*X*_(*c*). The theoretical ROC curve is a function $\text{ROC}(t)=1-F_{Y}[F_{X}^{-1}(1-t)]$, where *t* = 1 − *F*_*X*_(*c*), is 1-specificity. Hence, the ROC curve plots 1-specificity against the sensitivity calculated for different values of the threshold *c*. The area under this curve (AUC) measures how well the marker discriminates between the two groups involved and is given by *P*(*Y* >*X*). Note that these definitions are a consequence of the assumption that high values of the marker are associated with the experimental group.

The simplest non-parametric estimation method for the ROC curve involves using empirical cumulative distribution functions. The empirical cumulative distribution function is defined for any given value *c*, to be the observed percentage of sample values less than or equal to *c*. The resulting estimated ROC curve is an increasing step function on the unit square. The area under this curve is equal to the Mann-Whitney U-statistic and provides an unbiased non-parametric estimator for the AUC [17]. Bamber [29] showed that the AUC, when calculated using the trapezoidal rule, is equal to the Mann-Whitney U-statistic.

This method was performed using functions from the ROC library from Bioconductor.

### Arrow plot

Plotting OVL against AUC gives rise to a graph which we called *arrow plot*. In order to identify different kinds of differentially expressed genes, it is necessary to select appropriate cutoff points both for the AUC and OVL. Differentially expressed genes will have low values for the OVL, say less than 0.5. Up-regulated genes will correspond to AUC near 1, down-regulated genes will correspond to AUC values near 0, and special genes will have AUC values around 0.5. An algorithm to check for bimodality is added, where special genes are highlighted using different colors depending whether bimodality is verified in case or control group or both.

### TNRC and ABCR statistics

Parodi et al. [5] developed two new ROC based methods to identify differentially expressed genes that may correspond both to proper and to not proper ROC curves. TNRC (Test for Not-proper ROC Curves) is a test to identify not proper ROC curves and ABCR (area between the ROC curve and the rising diagonal) statistic represents a measure of the distance between the distributions of gene expression in two classes.

The ABCR statistic is obtained using the empirical ROC curve, where ties are not considered. In that sense, if *n*_0_ is the number of individuals observed with *X* (considering the same notation as in non-parametric AUC section) and *n*_1_ the number of individuals observed with *Y *, *n* = *n*_0_ + *n*_1_ will be the total of individuals observed and *m*_0_ ≤ *n* will represent the total observations without ties.

They first rank the genes accordingly to ABCR (5).

(5) $$\text{ABCR}=\overset{m_{0}}{\underset{k=1}{\sum }}|{\text{AUC}}_{k}-A_{k}|,$$

where *AUC*_*k*_ is the partial area under a ROC curve between the consecutive abscissa points for *k* = 1,…,*m*_0_ computed according to the standard trapezoidal rule, and $A_{k}=\frac{2k-1}{2m_{0}^{2}}$ represent the partial area of the chance line. The first *g* genes correspond to a False Discovery Rate defined by the user.

TNRC statistic is used to test for not proper ROC curves:

(6) $$\text{TNRC}=\overset{m_{0}}{\underset{k=1}{\sum }}|{\text{AUC}}_{k}-A_{k}|-|\text{AUC}-0.5|$$

where AUC is the area under the empirical ROC curve. Not proper ROC curves are identified by high values of the TNRC statistic.

## Competing interests

The authors declare that they have no competing interests.

## Authors’ contributions

CSF, a PhD student developed and implemented the method under the guidance of her advisors MAAT and LS. All authors read and approved the final manuscript.

## Supplementary Material

Additional file 1 R code for implementation of Algorithms 1 and 2. Click here for file

Additional file 2 Biological description of the 20 special genes selected in the Lymphoma data. Click here for file
