# Supplementary material for: Arrow plot: a new graphical tool for selecting up and down regulated genes and genes differentially expressed on sample subgroups
Source: BMC Bioinformatics. 2012 Jun 26;13:147. doi: 10.1186/1471-2105-13-147 (PMC3542259; doi:10.1186/1471-2105-13-147)
Supplement: Additional file 2 — Biological description of the 20 special genes selected in the Lymphoma data. [file 1471-2105-13-147-S2.pdf]

Gene name	Description
BCL7A	Chromosome 12q24.1 is the site of a recurrent breakpoint in high-grade B-cell non-Hodgkin lymphoma of the amino-terminus of *BCL7A* defined a new mechanism in the pathogenesis of a subset of high-grade B-cell non-Hodgkin lymphoma.
Immunoglobulin J chain	The J chain is found in pentameric IgM, and it is also important as a secretory immunoglobulin.
MAPKKK5	JUN N-terminal kinases (JNKs) are MAPKs that stimulate transcriptional activity of JUN in response to growth factors, proinflammatory cytokines, and certain environmental stresses.
Similar to KIAA0050	ACAP1 and ACAP2 were recruited to platelet-derived growth factor (PDGF)-induced dorsal membrane ruffles in NIH 3T3 mouse fibroblasts, and overexpression inhibited ruffle formation.
Histone deacetylase 3	Acetylation and deacetylation of histone proteins alters chromosome structure and affects transcription factor access to DNA.
VRK2 kinase	Genes involved in the regulation of cell division.
LR11	Is genetically associated with late-onset Alzheimer's disease and is thought to be involved in neurodegenerative processes.
BL34	Sequence of a human B-cell specific immediate early gene, that is inducible in response to several B-cell activation signals.
Placental bikunin	Is a potent inhibitor of serine proteases involved in blood coagulation and fibrinolysis such as human plasmin, plasma and tissue kallikrein, and factor XIa.
LBR	Encodes the lamin B receptor, an inner nuclear membrane protein that binds lamin B. Since the lamin B receptor may be a sterol reductase, loss of most *LBR* expression might lead to changes in sterol metabolism that cause developmental abnormalities.
Immunoglobulin kappa light chain	Small polypeptide subunit of an antibody. Antibodies are produced by B lymphocytes, each expressing only one class of light chain (Kappa or lambda).
BNIP3	Activates expression of the gene encoding NIP3, in turn primes cells for apoptosis under conditions of persistent oxygen deprivation.This pathway may play play a role in cell death resulting from cerebral and myocardial ischemia.
CLK3 kinase	A family of enzymes that catalyze the phosphorylation of proteins at serine and threonine residues.
DNA Ligase III	Seals DNA strand breaks that arise during the process of meiotic recombination in germ cells and as a consequence of DNA damage in somatic cells.
PARP	Is required for cellular repair. Inhibitors of this enzyme potentiate the lethal effects of noxious agents.
